# 

**Published:** 1852-10

**Authors:** 


☞ Dr. Maxson's communication, received too late for insertion in this No., will appear in our next.

*Notice.*—From the 15th of October till the last of February, next, the address of the editor of this Journal will be Louisville, Ky. Letters and communications may be directed to him at that place. With the aid of his colleagues and other friends, the publication of the Journal will be continued under his supervision, as heretofore.

---

☞ Notices of Transactions of several Medical Associations are crowded out of this No.

---

☞ We beg leave to remind our readers that the usual limits of the Journal are exceeded in this No. by sixteen pages.

